# *In vitro* growth of pneumococcal isolates representing 23 different serotypes

**DOI:** 10.1186/1756-0500-6-208

**Published:** 2013-05-23

**Authors:** Hans-Christian Slotved, Catherine Satzke

**Affiliations:** 1 Department of Microbiology and Infection Control, Statens Serum Institut, Copenhagen, Denmark; 2 Pneumococcal Research, Murdoch Childrens Research Institute, The Royal Children’s Hospital, Flemington Road, Parkville, Victoria, Australia; 3 Department of Microbiology and Immunology, The University of Melbourne, Parkville, Victoria, Australia

**Keywords:** Serum broth, Todd-Hewitt broth, *Streptococcus pneumoniae*

## Abstract

**Background:**

Although there are established methodologies for pneumococcal carriage studies, recent studies have extended these by conducting a culture amplification step prior to pneumococcal identification or serotyping. However, few data are available comparing the growth of different serotypes.

**Findings:**

We compared the growth of individual isolates representing 23 serotypes in serum broth, and Todd-Hewitt broth from four different manufacturers. Following overnight incubation of low inocula, there were differences in the final growth densities of individual isolates. These can be minimised with the use of optimal media.

**Conclusions:**

These data caution against using broth culture amplification of nasopharyngeal samples for some applications.

## Findings

*Streptococcus pneumoniae* (the pneumococcus) is a major cause of diseases such as pneumonia and meningitis, particularly in developing countries. At present, more than 90 immunologically-distinct capsular types have been described [1]. Several vaccines targeting pneumococcal capsular polysaccharide have been developed and licensed with the aim of reducing pneumococcal disease [2,3]. To monitor long-term vaccine effectiveness, it is likely that countries will monitor invasive pneumococcal disease and pneumonia [4,5], as well as changes to nasopharyngeal carriage, the latter being particularly relevant for resource-poor settings [6]. There are standard methods for assessing carriage [7], however broth culture amplification prior to pneumococcal identification and/or serotyping has also been described [8,9]. Many new methods, some of which are quantitative, are capable of detecting multiple serotypes in a swab sample [5]. However, the potential of different serotypes to grow at different rates, and thereby reduce the ability to accurately quantitate serotypes following culture amplification, remains insufficiently studied [10].

A previous study on testing and evaluating a serological test found that incubation in different growth media resulted in different average final growth densities [11]. Given the increased focus on individual serotypes, the current manuscript presents data from 23 different pneumococcal isolates, representing each of the serotypes in the current polysaccharide vaccine (23vPPS), following overnight incubation in five different media: Todd Hewitt (TH)-broth from four manufacturers (Oxoid, Sigma, Difco and SSI Diagnostica) and serum broth (SSI Diagnostica).

Isolates (sourced from the Streptococcus Unit, Statens Serum Institut, Denmark) were stored as lyophilized cultures, or in nutrient beef broth with 10% glycerine (SSI Diagnostica) at -80°C, and subcultured on 10% horse blood agar plates (HBA, SSI Diagnostica) prior to use. To assess growth, fresh overnight cultures of pneumococci (grown at 37°C in 5% CO_2_) were suspended in physiological saline to a concentration of ~1 × 10^8^ CFU/ml (Optical Density (OD)_540_ = 0.28 measured by a colorimeter (Sherwood)). Following dilution in physiological saline, 30 μl was inoculated into 3 ml broth (preheated to 37°C) resulting in a mean starting concentration of 1.21 × 10^3^ CFU/ml (95% CI: 7.09 × 10^2^, 1.72 × 10^3^ CFU/ml). Broths were incubated overnight at 37°C in 5% CO_2_. Viable counts were then conducted from serial dilution of a 100 μl aliquot in physiological saline plated on HBA. Results are pooled data from duplicate 18 and 24 h overnight incubations, resulting in n = 4 for each isolate in a given medium, except for eight missing data points spread across four media and four serotypes. Data were analysed using ANOVA with Bonferroni’s post-test or unpaired t-test as outlined below using GraphPad Prism version 5.0.

**Figure 1:**
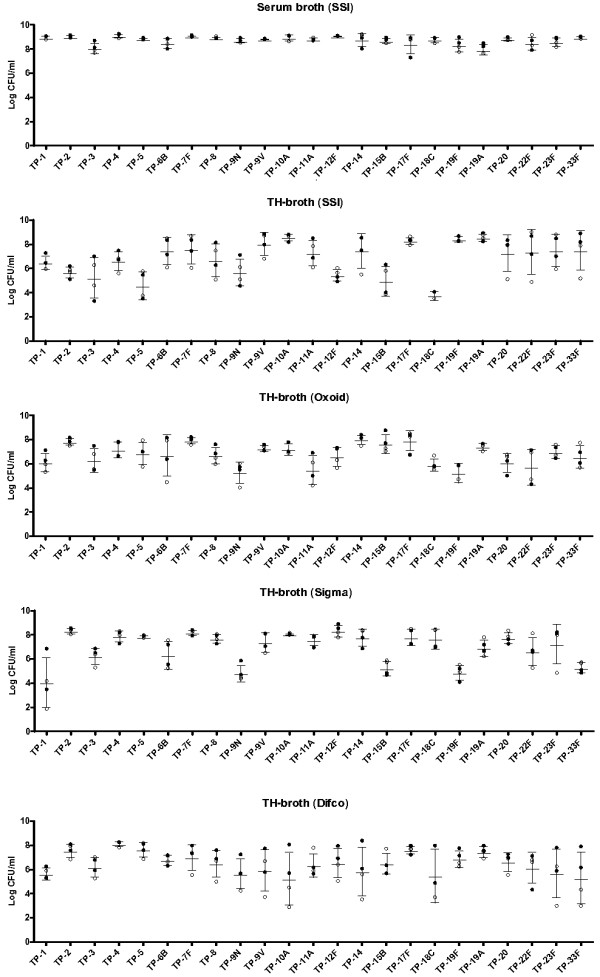
**Final growth densities of 23 isolates (representing each of the serotypes included in 23vPPS) following overnight incubation (18 h, closed circles or 24 h, open circles) in serum broth (SSI) or TH-broth (SSI, Oxoid, Sigma, Difco) with a starting inocula of approximately 1 × 10^3^ CFU/ml.** Horizontal lines are the mean +/- SD.

Figure 1 depicts the density for each of the 23 different pneumococcal isolates grown in five different broths following overnight incubation. As described in Slotved and Kerrn [11], serum broth generally resulted in superior growth compared with each of the TH-broths. Within each media type there was a significant difference in the final density of all serotypes using an ANOVA (p < 0.001), the exception being Difco (p = 0.05). Bonferroni’s post-test was used to compare the final growth densities between pairs of individual isolates when grown in the same media type. With serum broth, 10/253 (4%) of such comparisons showed significant differences (p < 0.05). TH-broth (SSI, Oxoid and Sigma) showed significant differences in 21, 8 and 69 (8%, 3% and 27%) of the 253 comparisons respectively. For TH-broth (Difco) we did not find significant differences in the growth of individual isolates, which may be attributable to large variation in the final densities for some serotypes. When we compared the individual isolates in TH-broth (Difco) with small standard deviations (SDs) (e.g. TP-1 versus TP-2, 4, 6B, 17F, 19F or TP-3 versus TP-4) there were significant differences using an unpaired t-test with Welch’s correction (p < 0.05), supporting this hypothesis. We also observed that, in general, the final densities of isolates grown in serum broth were less variable than when grown in the other media types.

To date, only a few studies have presented data showing the growth and survival of pneumococci of different serotypes *in vitro*[10,12-14]. Consistent with these, we found media-dependent differences in the growth of individual isolates representing 23 serotypes. Other studies have found that the metabolic demands of production of serotype-specific capsular polysaccharide can effect growth in restricted media, and also found that a serotype 18C strain showed a prolonged lag phase even in enriched media [10,15].

**Figure 2:**
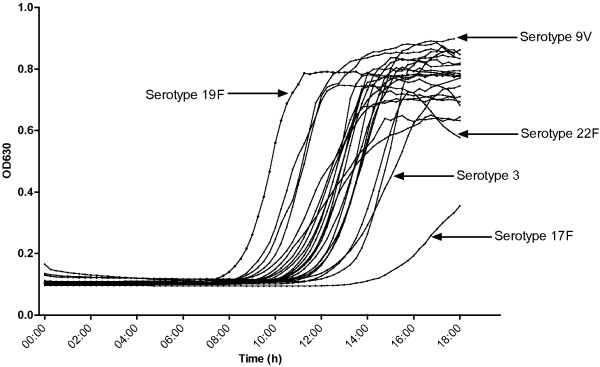
**Growth curves of 23 isolates (representing each of the serotypes included in 23vPPS) following incubation in serum broth (SSI).** 1 × 10^3^ CFU/ml (50 μl) were inoculated into 250 μl pre-warmed broth in a microtitre plate, and then incubated at 37°C in 5% CO_2_. The optical density (OD 630 nm) was measured every 15 min for 18 h using a spectrophotometer.

To explore growth of the 23 isolates further, we conducted preliminary growth curves in the five media types. For each isolate a 2 × 10^8^ CFU/ml physiological saline solution was made (based on a measured OD value of 0.2 at 600 nm). From this solution a dilution of 1 × 10^3^ CFU/ml was made, and 50 μl of this dilution was inoculated into 250 μl pre-warmed broth in a microtitre plate. Plates were incubated at 37°C in 5% CO_2_ and the optical density (at 630 nm) measured every 15 min for approximately 18 h using a spectrophotometer. Growth curves of some isolates differed. For example, in serum broth (Figure 2) the final density ranged from 0.36 (serotype 17F) to 0.90 (serotype 9V), and the mid-log point occurred at <10 h (serotype 19F) and ~15 h (serotype 3). Similar variation in final density and mid-log point were seen with the other media, although the lag time was generally longer, and as a result, lower final densities were observed (data not shown).

One limitation of this study is that isolates from a strain collection may have different growth characteristics than carriage isolates. We did not elucidate the mechanism for the observed differences in final growth densities (Figure 1). However, it is likely that differences occur in all phases of the growth curve, including in the levels of autolysis, consistent with Figure 2 (e.g. serotype 22F). Further experiments could compare the growth characteristics of isolates at shorter time-points, for example by conducting viable counts after 4-6 h incubation.

Our results confirm that different pneumococcal isolates grow to different densities in some media. On the basis of these results, we recommend avoiding broth culture amplification steps that utilise overnight incubation of nasopharyngeal samples prior to serotyping. Where this cannot be avoided, an enriched growth medium such as serum broth is likely to minimise the observed growth differences between isolates.

### Availability of supporting data

The data set(s) supporting the results of this article is (are) included within the article (and its additional file(s)).

## Abbreviations

TH-broth: Todd-Hewitt broth; SSI: Statens Serum Institut; 23vPPS: Pneumococcal polysaccharide vaccine PNEUMOVAX23®.

## Competing interests

CS has no financial competing interests. HCS is employed by the SSI which produces several of the products compared in this manuscript.

## Authors’ contributions

HCS carried out the experimental work. HCS and CS participated in the design of the study, performed the statistical analysis and wrote the manuscript. Both authors read and approved the final manuscript.
